# Supplementary material for: Significant Reversal of Facial Wrinkle, Pigmented Spot and Roughness by Daily Application of Galactomyces Ferment Filtrate-Containing Skin Products for 12 Months—An 11-Year Longitudinal Skin Aging Rejuvenation Study
Source: J Clin Med. 2023 Feb 1;12(3):1168. doi: 10.3390/jcm12031168 (PMC9917576; doi:10.3390/jcm12031168)

Supplementary Table S1 Chronological facial skin measurements (mean (S.D.)) in 1999 and 2010 (at 0, 2, 8, and 12M)

	Hydration	TEWL	Wrinkle	Spot	Roughness	Overall Skin Aging Score	Pore	Sebum	Lightness	Yellowness	Redness	Elasticity
1999	*56.71 (7.11)	*12.34 (1.83)	*0.203 (0.089)	*0.188 (0.059)	*0.728 (0.200)	*2.889 (0.905)	*0.0156 (0.068)	*36.72 (7.95)	*59.27 (1.35)	*20.39 (0.96)	12.55 (0.92)	*0.282 (0.026)
0 M (2010)	47.76 (8.68)	13.36 (1.18)	0.370 (0.142)	0.283 0.074)	0.864 (0.121)	4.001 (1.281)	0.0201 (0.089	38.68 (8.05)	57.60 (1.29)	21.63 (0.95)	12.43 (0.76)	0.242 (0.028)
2 M (2010)	*53.07 (9.24)	*12.78 (1.27)	*0.326 (0.135)	*0.236 (0.064)	*0.81 (0.188)	*3.528 (1.185)	*0.0173 (0.075)	*36.7 (8.82)	*58.10 (1.44)	21.66 (0.93)	12.12 (0.74)	*0.264 (0.027)
8 M (2010)	*59.61 (10.08)	*12.48 (1.79)	*0.308 (0.137)	*0.209 (0.063)	*0.788 (0.199)	*3.286 (1.058)	*0.0159 (0.065)	*36.79 (9.95)	*58.55 (1.25)	21.27 (0.84)	12.47 (0.59)	*0.279 (0.024)
12 M (2010)	*57.64 (9.84)	*12.01 (1.31)	*0.232 (0.126)	*0.197 (0.067)	*0.762 (0.196)	*3.068 (0.980)	*0.0165 (0.071)	*36.19 (10.09)	*59.50 (1.19)	*20.50 (0.86)	*11.37 (0.77)	*0.286 (0.027)

All 86 subjects were treated with twice-daily application of G3 product formulas for 12M (0M to 12M).

*: $P<0.05$ compared to the measurements at 0M (2010))

Supplementary Figure S1 G3 products exerted the potent anti-aging effects against wrinkles, spots and roughness similarly in subjects of different age groups (30s through 50s or more). The overall skin aging score was also significantly reversed by G3 products in different age groups.

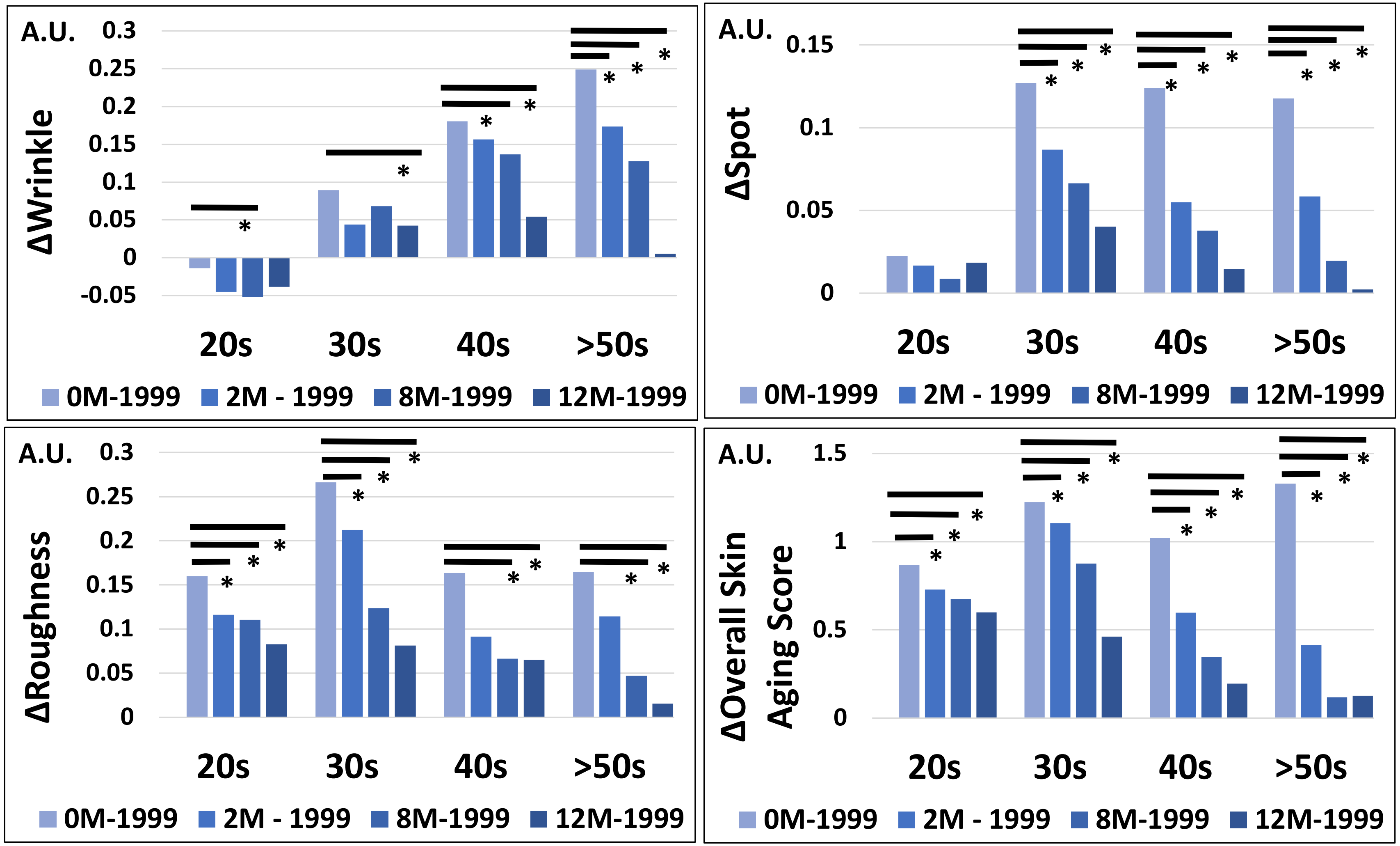

Supplement: Supplementary file 1 [file jcm-12-01168-s001.zip › jcm-2142511-supplementary.pdf]
